# Feasibility, reproducibility, and cold-induced energy expenditure using whole-room calorimetry in adults and children

**DOI:** 10.1210/jendso/bvag102

**Published:** 2026-05-05

**Authors:** Paige Cheveldayoff, Bader Alamri, Dongdong Wang, Rogelio Cruz Gonzalez, Aaron C Q Thomas, Norm Konyer, Michael D Noseworthy, Hertzel C Gerstein, Zubin Punthakee, Gregory R Steinberg, Katherine M Morrison

**Affiliations:** Centre for Metabolism, Obesity and Diabetes Research, McMaster University, Hamilton, ON, Canada L8N 3Z5; Department of Pediatrics, McMaster University, Hamilton, ON, Canada L8N 3Z5; Centre for Metabolism, Obesity and Diabetes Research, McMaster University, Hamilton, ON, Canada L8N 3Z5; Division of Endocrinology, Department of Medicine, McMaster University, Hamilton, ON, Canada L8N 3Z5; Centre for Metabolism, Obesity and Diabetes Research, McMaster University, Hamilton, ON, Canada L8N 3Z5; Division of Endocrinology, Department of Medicine, McMaster University, Hamilton, ON, Canada L8N 3Z5; Centre for Metabolism, Obesity and Diabetes Research, McMaster University, Hamilton, ON, Canada L8N 3Z5; Department of Pediatrics, McMaster University, Hamilton, ON, Canada L8N 3Z5; Centre for Metabolism, Obesity and Diabetes Research, McMaster University, Hamilton, ON, Canada L8N 3Z5; Department of Pediatrics, McMaster University, Hamilton, ON, Canada L8N 3Z5; Imaging Research Centre, St. Joseph′s Healthcare Hamilton, Hamilton, ON, Canada L8P 3B7; Centre for Metabolism, Obesity and Diabetes Research, McMaster University, Hamilton, ON, Canada L8N 3Z5; Imaging Research Centre, St. Joseph′s Healthcare Hamilton, Hamilton, ON, Canada L8P 3B7; Electrical and Computer Engineering, McMaster University, Hamilton, ON, Canada L8N 3Z5; Centre for Metabolism, Obesity and Diabetes Research, McMaster University, Hamilton, ON, Canada L8N 3Z5; Division of Endocrinology, Department of Medicine, McMaster University, Hamilton, ON, Canada L8N 3Z5; Centre for Metabolism, Obesity and Diabetes Research, McMaster University, Hamilton, ON, Canada L8N 3Z5; Division of Endocrinology, Department of Medicine, McMaster University, Hamilton, ON, Canada L8N 3Z5; Centre for Metabolism, Obesity and Diabetes Research, McMaster University, Hamilton, ON, Canada L8N 3Z5; Division of Endocrinology, Department of Medicine, McMaster University, Hamilton, ON, Canada L8N 3Z5; Centre for Metabolism, Obesity and Diabetes Research, McMaster University, Hamilton, ON, Canada L8N 3Z5; Department of Pediatrics, McMaster University, Hamilton, ON, Canada L8N 3Z5

**Keywords:** pediatrics, energy expenditure, indirect calorimetry, obesity, cold exposure, BAT activity

## Abstract

**Context:**

To understand energy balance, whole-room indirect calorimetry (WRIC) allows for accurate measurement of energy expenditure (EE).

**Objective:**

To examine the relationship between cold-induced resting EE and brown adipose tissue (BAT) activity measured by magnetic resonance imaging (MRI) and to evaluate WRIC system (WRICS) performance and feasibility of use in children and adults.

**Methods:**

The WRICS was equipped with a Promethion High-Definition Room Calorimetry System. Technical validation utilized nitrogen (N_2_) and carbon dioxide (CO_2_) gas infusions. Healthy adults (n = 21) and children aged 8-17 years (n = 17) attended two 4-hour WRIC visits (one week apart) and one MRI visit. Resting EE at 25 °C (REE_25_) was compared between visits and to REE at 18 °C (REE_18_). Recruitment and completion rates were examined. BAT activity was assessed by MRI as the decline in supraclavicular proton density fat fraction during 18 °C cold exposure.

**Results:**

Gas infusion testing confirmed high accuracy (respiratory exchange ratio [RER] = 0.99; 95% CI 0.991-0.996). Study completion rates were high (adults: 20/21; children: 17/18). REE_25_ over a 10-minute period was consistent between visits (adults: 1.68 ± 0.462 vs 1.66 ± 0.301 kcal/min, *P* = 0.77; children: 1.50 ± 0.358 vs 1.58 ± 0.348 kcal/min, *P* = .25). Cold exposure increased fasting EE by 0.21 kcal/min (adults) and 0.14 kcal/min (children). BAT activity was correlated with REE_18_ in adults (*r* = 0.49, *P* = .04).

**Conclusion:**

WRICS use was feasible in adults and children. Changes in EE during cold (ie, cold-induced thermogenesis) were measurable and related to BAT activity, supporting the usefulness of this system in the assessment of EE in response to interventions in adults and children.

Obesity is a common condition that contributes to considerable morbidity and mortality [1, 2]. Energy imbalance resulting from the disparity between energy intake, absorption, and expenditure is a key factor in the development of obesity and its accompanying metabolic complications, including metabolic dysfunction–associated steatotic liver disease (MASLD) and type 2 diabetes [3].

Total energy expenditure (EE) is comprised of resting energy expenditure (REE), diet-induced thermogenesis (DIT), and physical activity EE, which includes both exercise- and nonexercise-related EE. REE is the amount of energy that the body requires for essential organ and cellular function when lying in a state of physiological and psychological rest. DIT, also known as the thermic effect of food, is energy expended for the digestion, absorption, and storage of food. REE accounts for 60% of total EE, while DIT and physical activity EE contribute 10% and 15% to 30%, respectively [4]. Critically, EE has been noted to decline with weight loss to a greater extent than predicted by changes in body composition alone—a phenomenon termed *metabolic adaptation to weight loss* or *adaptive thermogenesis* [5]. Given the potential contribution of metabolic adaptation to weight regain, understanding of the mechanisms contributing to this must be better understood—but requires accurate, reproducible measurement of EE.

It has long been known that energy expenditure can be quantified by measuring respiratory gas exchange, known as indirect calorimetry [6]. Whole-room indirect calorimetry (WRIC) allows study participants to move freely in the room and can be used over hours to days, enabling measurement of all components of EE. Furthermore, improvements in technology have enhanced the sensitivity of whole-room calorimeters, thereby reducing response times to minutes. To enhance comparability of findings from multiple laboratories, an expert panel established the Room Indirect Calorimetry Operating and Reporting Standards, version 1.0 (RICORS 1.0) in 2019 to establish minimum requirements for reporting technical specifications, data reduction and analytical approaches, performance standards, elements of study design, and outcome measures for studies employing WRIC [7]. These standards will enable comparison of findings relating to human energy metabolism across sites, thereby enhancing knowledge in this important field.

REE is influenced by age, body composition, diet, environmental temperature, biological sex, hormone levels, and medications [4, 8-10]. One contributor to variation in REE is brown adipose tissue (BAT) activity through nonshivering thermogenesis [11]. As BAT activity is linked to better metabolic health in adults [12-14] and in children [15], there has been a growing interest in understanding factors that contribute to or inhibit BAT activity across the life course. The stimulation of BAT by cold exposure is fundamental to measurement of BAT activity. Furthermore, BAT activity is influenced by pharmacotherapy and is regulated by the sympathetic nervous system and hormones.

To better understand EE in adults and children, the Centre for Metabolism, Obesity and Diabetes Research at McMaster University has established 2 new whole-room indirect calorimeters. Here we report the technical specifications and performance standards of this new system in the evaluation of REE at 25 °C and during an 18 °C cold exposure in adults and children. We discuss the feasibility of using WRIC in children, the ability of the system to detect changes in REE during cold exposure (REE_18_) and after eating a standardized mixed meal. Further, we examine the relationship of cold-induced changes in EE and a magnetic resonance imaging (MRI)-based measurement of BAT activity in both adults and children.

## Methods

### System accuracy of the whole-room indirect calorimetry system

This technical validation complies with the RICORS 1.0 recommendations for initial validation of the 2 whole-room indirect calorimetry systems (WRICS) at the Centre for Metabolism, Obesity and Diabetes Research, McMaster University. The rooms, measuring 3.5 m W × 2.9 m D × 2.4 m H (volume 23.9 m^3^) and 2.3 m W × 2.9 m D × 2.4 m H (volume 15.6 m^3^) (Cantrol Environment Systems Inc, Toronto, Canada), were constructed at McMaster University in Hamilton, Ontario, within the McMaster University Medical Centre. The larger calorimeter, which was used for the current study, is equipped with a sofa bed, chair, table, entertainment station, sink, and toilet for participant use. In addition, both rooms are equipped with windows, an intercom for communication, and a dual-lever airtight lock for food or water delivery to participants while in the room.

Respiratory exchange within the chamber, as well as room temperature and humidity, were measured using the Promethion GA-3m2/FG-250 model (Promethion High-Definition Room Calorimetry System, Sable Systems International, NV, USA). The manufacturer's guidelines were adhered to with respect to daily and periodic calibration. Concentrations of CO_2_ and O_2_, as well as water vapor pressure and barometric pressure, were measured by the system (flow rate 250 L/min). These data were analyzed using the internal software from Sable Systems International (Macrointerpreter version 26.1, macro version 1.5.7). Gas signal concentrations and kcal data were smoothed using a single moving average (boxcar) filter with a 30-minute window. This software also calculated glucose and fatty acid oxidation rates using Frayn's equation [16] and energy expenditure using the Weir formula [17].

The system performance portion of the technical validation of the room calorimeters was conducted using the gas infusion method specified in RICORS 1.0 [7]. High-purity N_2_ (99.1%) and CO_2_ (99.99%) gases (LINDE Canada Inc, Scarborough, Canada) were infused at specific rates using a calibrated mass-flow controller for each respiratory exchange ratio (RER). For an RER of 1.0, CO_2_ was infused at 0.2500 L/min, and N_2_ at 0.9434 L/min. For an RER of 0.8, CO_2_ was infused at 0.2000 L/min, with N_2_ also at 0.9434 L/min. Additionally, in compliance with RICORS 1.0 “zero” or empty room, tests were performed without gas infusion. To ensure the absence of any leaks in the system, standard recovery tests were performed monthly.

### Feasibility and reproducibility of measurement in adults and children

This was a 3-visit, cross-sectional study of adults and children and was undertaken from October 2022 to August 2024. Participants between the ages of 8 and 40 years who were able to communicate in English were included. Exclusion criteria, ascertained during a prescreen and confirmed at the baseline visit, included a history of type 1 or 2 diabetes, prior bariatric surgery or liver transplantation, current pregnancy or breast feeding, contraindications for MRI (claustrophobia, implanted metal, metallic injuries, recent tattoo or weight >136 kg) and current use of any of several medication classes including (antihyperglycemics, antidepressants, antipsychotics, anxiolytics, beta-blockers, beta-adrenergic agonists, and corticosteroids). Details are shown in Table S1 [18]. Recruitment occurred through advertisement locally and in the community. Feasibility was evaluated by assessing study recruitment and visit completion, with minimal attrition (<15%) indicating feasibility. The study was approved by the Hamilton Integrated Research Ethics Board (HiREB), and all participants either provided informed consent or a parent/guardian provided informed consent and the participant provided assent at the initial visit.

Each of the 3 study visits (2 EE/metabolic visits in the larger WRICS and 1 imaging visit) occurred with the participant fasting for at least 8 hours. The participants were instructed to refrain from ingesting caffeine for at least 12 hours, to refrain from physical activity for 48 hours and to abstain from foods high in serotonin (nuts, avocados, tomatoes, bananas, kiwis, pineapple, and plums) for at least 24 hours prior to all study visits, due to potential inhibitory effects on BAT activity [19, 20]. Anthropometrics, body composition, and energy expenditure were evaluated at the EE/metabolic visit and questionnaires including demographics and health were completed at the time of the imaging visit. Participants completed a 3-day food log and a questionnaire pertaining to participant demographic information (age, sex, ethnicity, and current medications) and personal/family health history. Pediatric participants completed a self-assessment of pubertal status [21].

### Energy expenditure and metabolic testing visits

The 2 EE/metabolic visits were conducted 1 week apart. Prior to EE measurement in the WRICS, anthropometry (height, weight, waist circumference), blood pressure, heart rate, and body composition were measured. Height was measured using a wall-mounted stadiometer (Seca, model 24) to the nearest 0.1 cm. Weight was measured using a calibrated electronic scale (InBody 570) to the nearest 0.1 kg. Waist circumference was measured at the midpoint between the lowest rib and iliac crest using a weighted measuring tape (OHAUS Pull Type Spring Scale, Parsippany, NJ, USA) set at 750 g to the nearest 0.1 cm. Height, weight, and waist circumference were measured 3 times and their respective averages were calculated. Blood pressure and heart rate were measured following a period of rest where the participants were instructed to sit quietly in an upright supported chair, with their legs uncrossed and to refrain from talking (One Piece Cuff—Suntech, CT40, Morrisville, NC, USA). Blood pressure and heart rate were measured 5 times for optimal accuracy and then averaged. Body mass index (BMI) was calculated using the averaged heights and weights (kg/m^2^), and, for children, BMI z-score was determined utilizing the standards set by the World Health Organization (WHO) [22]. Body composition was measured at one visit using dual-energy x-ray absorptiometry (DXA, Lunar PRODIGY Advance 8743; GE, Healthcare, Waukesha, WI). The Mostellar equation (1987) [23] was used to calculate body surface area (m^2^).

**Figure 1:**
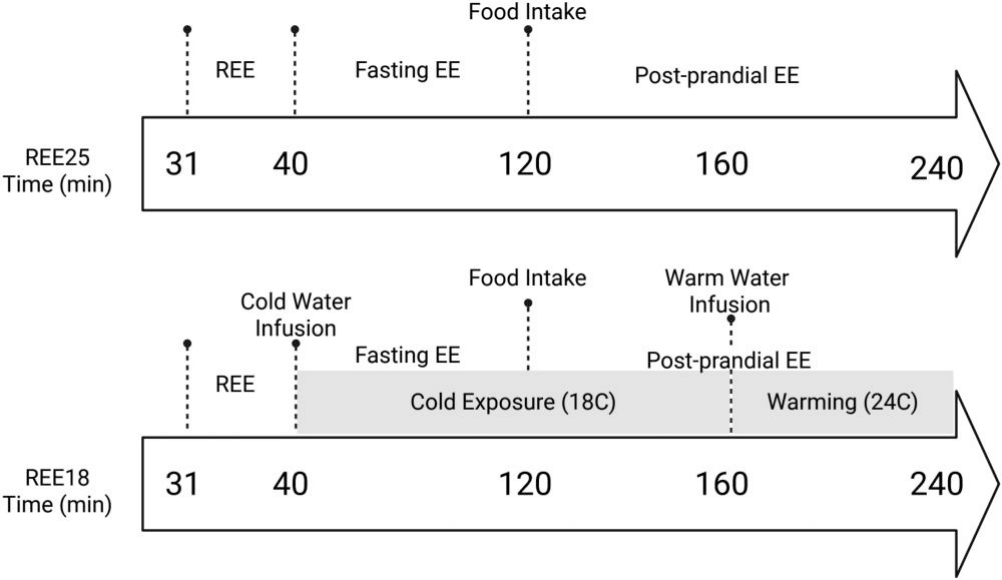
Metabolic/energy expenditure visit schemata at room temperature and with cold.

At both WRICS study visits, participants wore a total body water-perfused suit (Med-Eng, Ottawa, Ontario, Canada). Participants were randomized to the order of REE measurement at either 25 °C (no water perfusion) or 18 °C (suit connected to a flow-controlled circulating bath set at 18 °C, located outside the WRICS and with a flow rate of 1 L/min, (Isotemp 6200 R28; Fisher Scientific, Waltham, MA, USA). These visits were conducted in a standardized manner (Fig. 1). Participants were seated quietly and awake for an initial 30-minute calibration period, and during the following 10-minute period of rest for measurement of REE, prior to application of the temperature conditions. To ensure consistent cold exposure, thermocouples (TMQSS-020G-2; OMEGA Engineering, Stamford, CT) fixed to the inlet and outlet manifolds enabled measurement of water temperature and data were recorded and logged at 15 seconds intervals with a data logger (PowerLab; ADInstruments, Sydney, Australia).

After the 2 hours of cold exposure, participants underwent a warming period (24 °C water) that lasted until the end of their time in the room. A standardized mixed meal comprised of 235 mL Ensure Nutrition Supplement (Abbott Nutrition, Chicago, Il, USA) and a 68 g CLIF Bar (Mondelez International) (Total: 475 kcal, 74 g carbohydrate, 22 g protein, 12 g fat) was provided at 120 minutes at both visits. REE for evaluating repeatability was measured from minute 31 to 40, fasting EE at either 25 °C or 18 °C, glucose oxidation and fatty acid oxidation from minute 41 to 120, and postprandial EE, glucose oxidation and fatty acid oxidation from minute 121 to -240 (*see* Fig. 1). Following ingestion of the meal, diet-induced thermogenesis was measured over a period of 2 hours and was calculated as the difference between preprandial EE and postprandial EE. After 4 hours in the room, participants completed a satiety questionnaire, exited the WRIC, and completed postprandial blood collection.

### Imaging visit

#### MRI studies

One week prior to the first EE/metabolic visit, BAT activity measurement was undertaken at the Imaging Research Centre at St. Joseph's Healthcare Hamilton (Hamilton, ON, Canada). This involved evaluation of cold-induced changes in the proton density fat fraction in the supraclavicular area with MRI as previously reported [15, 24]. Briefly, after a 30-minute acclimation period at room temperature, participants underwent the baseline MRI scan with a 3-Tesla whole-body scanner (Discovery MR750; GE Healthcare, Waukesha WI). They were then fitted with the same water-perfused suit and flow-controlled circulation bath at 18 °C as described above. During the 1-hour cold exposure, the participants lay on a hospital stretcher in a semi-reclined position and were allowed to access their personal or provided devices for movies or music but were not permitted to engage in any movement activities. Measurement of inlet and outlet manifolds was completed as done for EE/metabolic visit, and participants self-reported any shivering. Room temperature and humidity were recorded using a Wireless Forecast Station with Pressure History (model no. WS-9037U-IT; La Crosse Technology, La Crosse, WI).

The MRI protocol and postimaging analysis were conducted as previously described [15]. Briefly, the proton density fat fraction (PDFF) was obtained using the iterative decomposition of water and fat with echo symmetry and least-squares estimation (IDEAL-IQ) pulse sequence. The PDFF is based on the ratio of the total density of mobile fat protons to the total density of water and mobile fat protons [25]. The PDFF was acquired using a head/neck/spine coil, with an axial image field covering the region between the C2/C3 vertebral disc and the T4/T5 disc (slice thickness 3 mm, 50 slices, flip angle 4°, echo time (TE) 1.3 ms, repetition time (TR) 8.4 ms, field of view (FOV) 340 mm, image resolution 1.52 × 1.42 × 3 mm, acceleration factor 2, and scan time 2.4 minutes). Six images were generated using this protocol: water-only, fat-only, in-phase, out-of-phase, corrected PDFF, and R2 images.

Postimaging analysis was conducted as previously reported using the AnalyzePro software (version 14; Biomedical Imaging Resource, Mayo Clinic, AnalyzeDirect, Overland Park, KS) [26]. The adipose tissue in the supraclavicular (SCV) region, between the C5-C6 and T1-T2 disc, was segmented using the sternocleidomastoid, trapezius, and clavicle as medial, posterior, and inferior landmarks, respectively. Regions of interest (ROIs) were drawn manually. A fat mask and T2 mask (2 to 25 ms) were applied as previously reported—to identify fat tissue (30%-100% fat fraction threshold) and to differentiate BAT from white adipose tissue and muscle. The voxels meeting the above specifications were averaged and reported as SCV-PDFF. To correct for partial volume effects, a two-dimensional erosion (1 × 3 voxels) was applied. The percent decline in PDFF (%) is a measure of BAT activity and is calculated as: [(precold SCV-PDFF − postcold SCV-PDFF)/(precold SCV-PDFF)] × 100.

### Statistical analysis

WRIC data were exported with Sable Systems MacroInterpreter (version 26.1) and Macro 1.5.7. Data analysis was conducted using SPSS statistics (version 29.0) and graphs were constructed using GraphPad Prism 10 (version 10.2). Data are presented as mean ± SD. Descriptive statistics including mean, SD, and CI calculations were used to examine the technical validation of the WRICS. Data were tested for normality using Shapiro-Wilk test and inspection of Q-Q plots. After confirming normal distribution, two-tailed paired *t* tests were used to compare within-participant differences in EE and respiratory exchange ratio (RER) and to compare fasting and postprandial fat oxidation and glucose oxidation, and diet-induced thermogenesis between the 25 °C and 18 °C visits. One-tailed paired *t* tests were used to compare fatty acid oxidation and glucose oxidation between the fasting and postprandial states. Pearson correlation coefficient was used to assess the relationship between BAT activity and REE_18,_ and correlations between body composition components (body fat %, fat mass index, lean mass, and lean mass index) and pre- and postprandial energy expenditure in both temperature conditions.

## Results

### Technical validation of the whole-room indirect calorimeters

Using the gas infusion method, the system calculated RER as 0.99 ± 0.001 (95% CI 0.991-0.996) for the larger room, and 0.98 ± 0.001 (95% CI 0.980-0.985) for the smaller room. When the rate of CO_2_ infusion was changed to 0.2000 standard liters per minute (SLPM) to simulate an RER of 0.8, the calculated RER was 0.82 ± 0.021 (95% CI 0.783-0.865, n = 7) and 0.809 ± 0.021 (95% CI 0.801-0.816, n = 4) for the larger and smaller rooms, respectively. To confirm the system's accuracy, empty room tests with an expected RER of 0.0 were conducted, and the system calculated RER as −0.04 ± 0.070 (95% CI −0.019-0.011) for the larger room and −0.04 ± 0.061 (95% CI −0.019-0.012) for the smaller room. Recovery tests were conducted monthly and calibrations were performed every 2 weeks to ensure the integrity and accuracy of the data collected. These tests involved measuring the amount of CO_2_ present after injecting CO_2_ gas at a rate of 0.2000 L/min overnight. The measured CO_2_ was then divided by the infusion rate and multiplied by 100 to determine the recovery rate. Recovery rates completed in the larger calorimeter throughout the JOULE study period (ie, November 2022 to August 2024) averaged 93.3 ± 4.97% (95% CI 90.6-96.9, n = 12).

These technical validation results demonstrate the accuracy and reproducibility of the data obtained, indicating that the system possesses a robust structure suitable for conducting human studies. All of the clinical studies described below were conducted using the larger of the 2 rooms.

### Feasibility and reproducibility of EE measurement in adults and children

**Figure 2:**
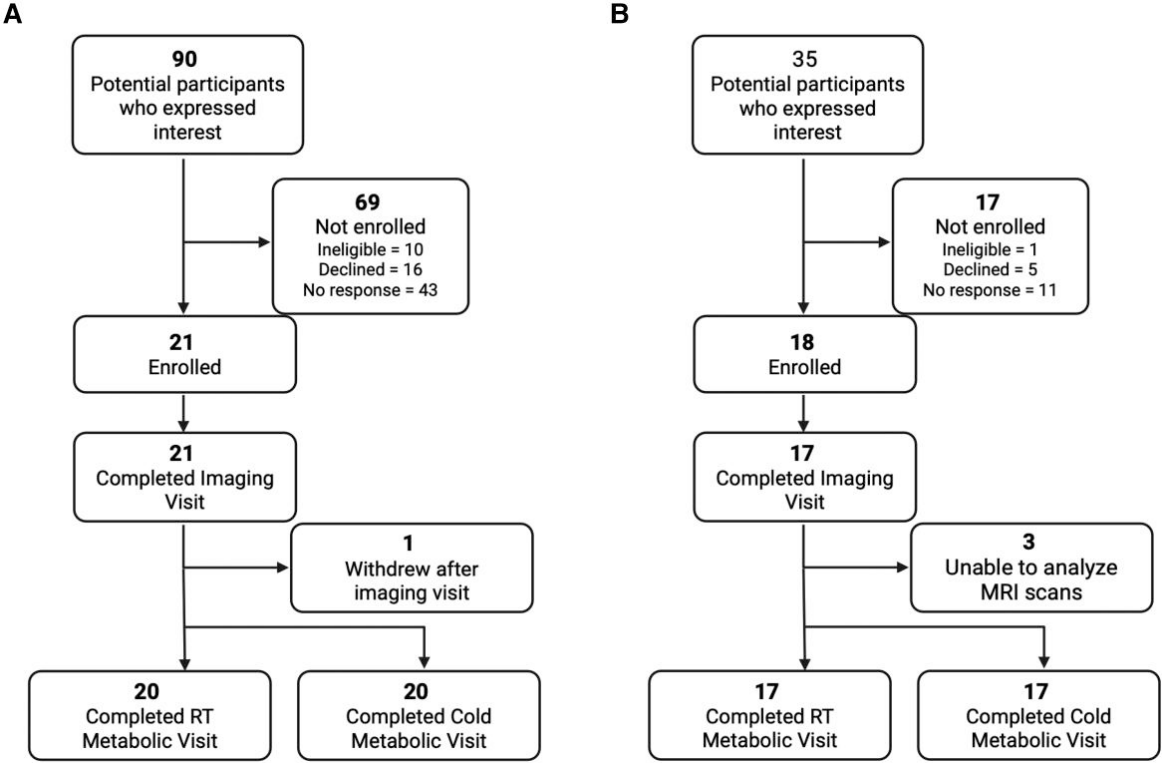
Participant recruitment and study completeness information in (A) adults and (B) children and adolescents.

Of the 90 adults and 35 children who expressed interest in the study, 21 adults and 18 children met the entry criteria and were enrolled (Fig. 2). One adult participant withdrew before completing the study and one pediatric participant had incomplete data. These 2 participants have been excluded from analysis.

The 20 adult (10 female; mean age 24.2 ± 5. 6 years) and 17 pediatric (6 female; mean age 13.2 ± 2.83 years) participants ranged in age from 8 to 36 years (*see* Table 1). Three adult participants (all male) had a BMI ≥ 30 kg/m^2^ and 8 adult participants (3 female, 5 male) had a BMI between 25 and 30 kg/m^2^; thus, 11/20 were classified as having overweight or obesity. Among the pediatric cohort, 3 participants were classified as having obesity and 2 as having overweight based on WHO BMI z-score for age [22]. Four pediatric participants were classified as prepubertal according to the self-assessed puberty staging questionnaire.

**Table 1:** Characteristics of the study participants

Variable name (units)	Adult	Pediatric
Sample size (n)	20	17
Age (y)	24.2 ± 5.56 (18, 36)	13.2 ± 2.83 (8, 17)
Sex (M/F)	10/10	11/6
Ethnicity (n)	Caucasian (n = 9) South Asian (n = 6) Middle Eastern (n = 5)	Caucasian (n = 14) African (n = 2) South Asian (n = 1)
Tanner puberty stage	N/A	3.2 ± 1.4 (1, 5)
Days between EE visits	8.25 ± 5.8 (2, 30)	7.35 ± 5.9 (3, 28)
Height (cm)	169.9 ± 7.8	161.7 ± 17.4
Weight (kg)	74.7 ± 15.9	57.4 ± 21.6
Waist circumference (cm)	61.9 ± 19.3	57.4 ± 14.3
Waist/height ratio	0.39 ± 0.06 (0.29, 0.52)	0.35 ± 0.06 (0.26, 0.47)
BMI (kg/m^2^)	25.6 ± 3.7 (20.2, 32.1)	21.1 ± 4.98 (15.8, 31.6)
BMI z-score (WHO) [21]	N/A	0.38 ± 0.89 (−0.54, 2.15)
Lean mass index (kg/m^2^)	17.52 ± 2.90 (13.2, 22.2)	14.4 ± 2.26(10.8, 17.7)
Total body fat (%)	27.8 ± 8.4 (13.6, 45.3)	26.1 ± 12.2 (6.7, 50.6)
Systolic BP (mmHg)	114.2 ± 9.6	107.5 ± 9.6
Diastolic BP (mmHg)	69.6 ± 7.9	66.1 ± 8.2
Heart rate	68.7 ± 11.5	76.3 ± 10.6

Units are mean ± SD (range) unless otherwise noted.

Abbreviations: BMI, body mass index; BP, blood pressure; EE, energy expenditure; WHO, World Health Organization.

### Reproducibility of REE

**Figure 3:**
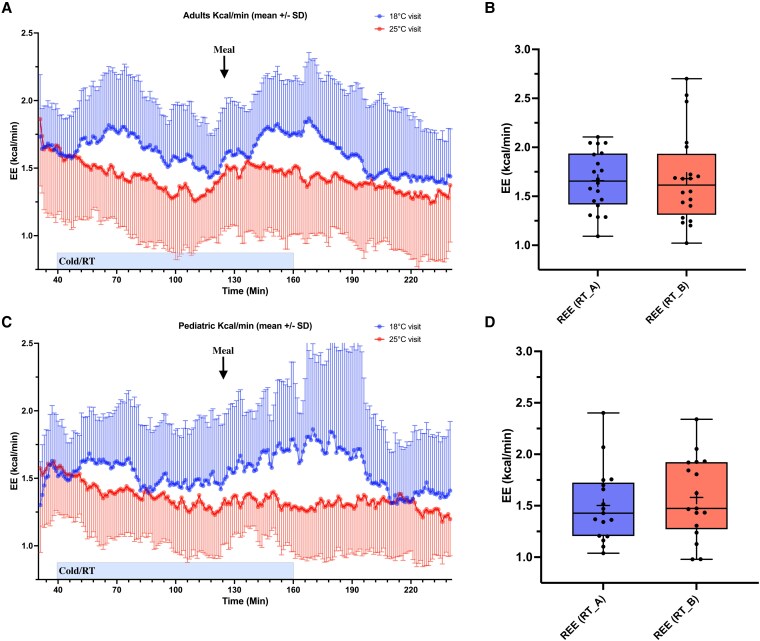
Whole visit calorie per minute data for (A) adult and (C) pediatric participants. Data are shown as mean ± SD. Cold (18 °C) H_2_O infused from minute 40 to minute 160 with a meal provided at minute 120. Resting energy expenditure at room temperature (REE RT) for (B) adults and (D) pediatric participants measured from minutes 31 to 40 during both study visits prior to Cold (18 °C) H_2_O infusion. Data shown as box and whisker with min and max values, cross denotes the group mean.

The EE for the entire visit for adults and for children is presented in Fig. 3. After the acclimation period, REE_25_ was measured between 31 and 40 minutes at each of the 2 EE/metabolic visits that occurred 7.3 days apart (3-31 days). As noted in Fig. 3B and Table S2 [18], REE was similar between visits in both adults; 1.68 ± 0.462 vs 1.66 ± 0.301 kcal/min (*P* = 0.77) and children; 1.58 ± 0.393 vs 1.50 ± 0.358 kcal/min, (*P* = .25). Similarly, there were no differences in the RER, room temperature, or room humidity between the 2 visits in either adults or children (see Table S2 [18]).

### EE at 18 °C or 25 °C

After the initial 40-minute period at 25 °C, participants then either continued at 25 °C or experienced a 2-hour period at 18 °C (10 adults and 5 children were randomized to experience the cold exposure at their first metabolic/EE visit, the remainder at their second visit)

**Figure 4:**
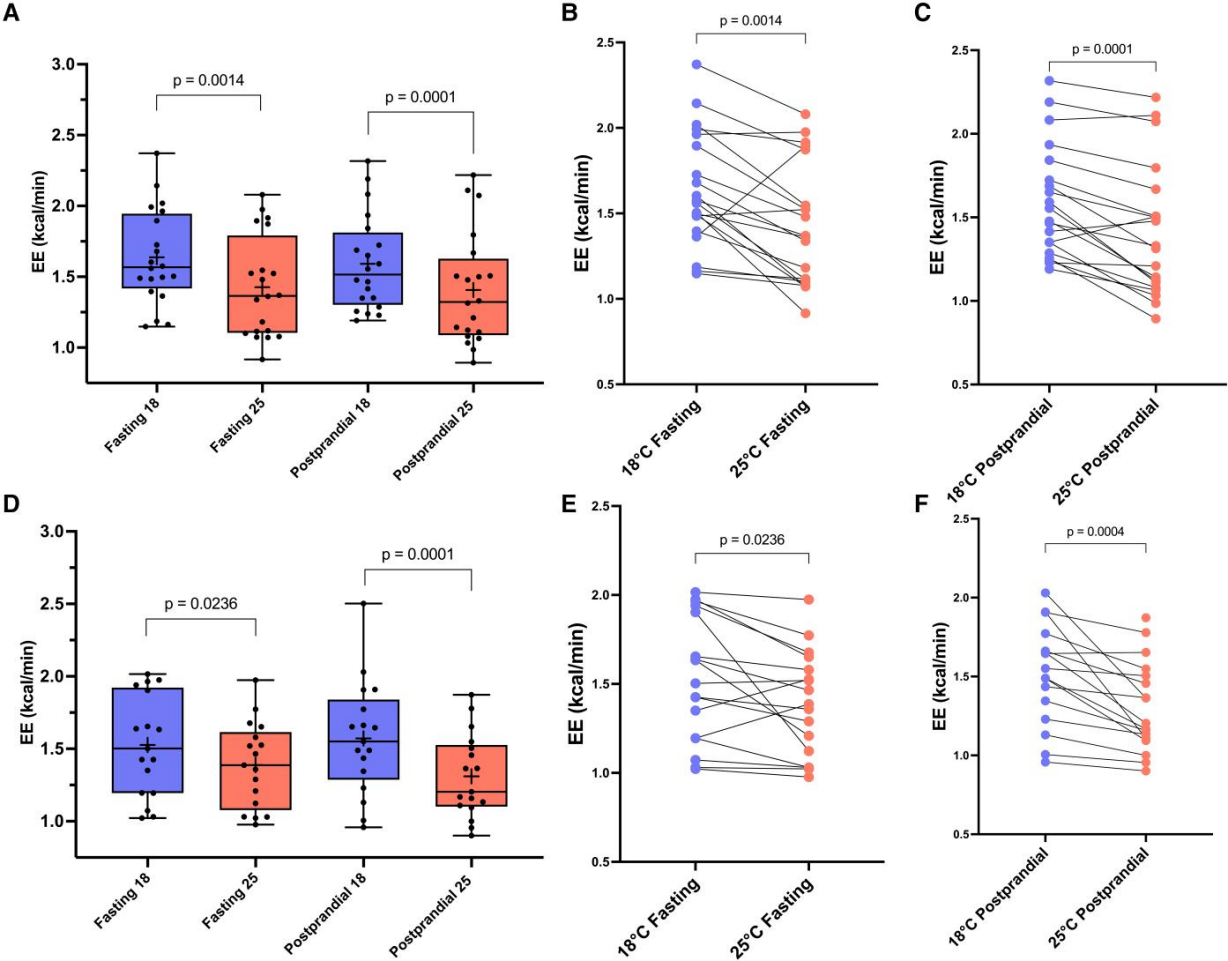
Fasting and postprandial EE (kcal/min) for (A-C) adult and (D-F) pediatric participants. A & D data shown as box and whisker with min and max values, cross denotes the group mean. B, C, E, F show within-participant differences between EE_18_ and EE_25_ for the fasting and postprandial timepoints.

Fasting REE_18_ was 14.7% higher in adults (Fig. 4A and 4B  *P* = <.0001) and 9.9% higher in children (Fig. 4D and 4E, *P* < .05) than fasting REE_25_*)*. Similarly, postprandial REE_18_ was higher in both adults (Fig. 4A and 4C; 13.1% higher, *P* = .001) and children (Fig. 4D and 4F; 19.9% higher, *P* < .001) in comparison to postprandial REE_25_. Postprandial EE was not higher than fasting at either temperature condition, in either adults or children (Fig. 4A and 4D).

### EE and body composition

As expected, lean mass was directly correlated with both pre- and postprandial EE in adults, under both temperature conditions (preprandial: EE_25_, *r* = 0.89, *P* < .0001; EE_18_, *r* = 0.79, *P* < .0001 and postprandial: EE_25_, *r* = 0.89, *P* < .0001; EE_18_, *r* = 0.78, *P* < .0001). Similarly, both lean mass and lean mass index (lean mass/height^2^) were directly correlated to preprandial and postprandial EE in children. REE was weakly directly related to REE_25_ in adults, but no other relationships between body fat percentage or fat mass index in adults or children were present.

### Influence of cold exposure on substrate utilization

**Figure 5:**
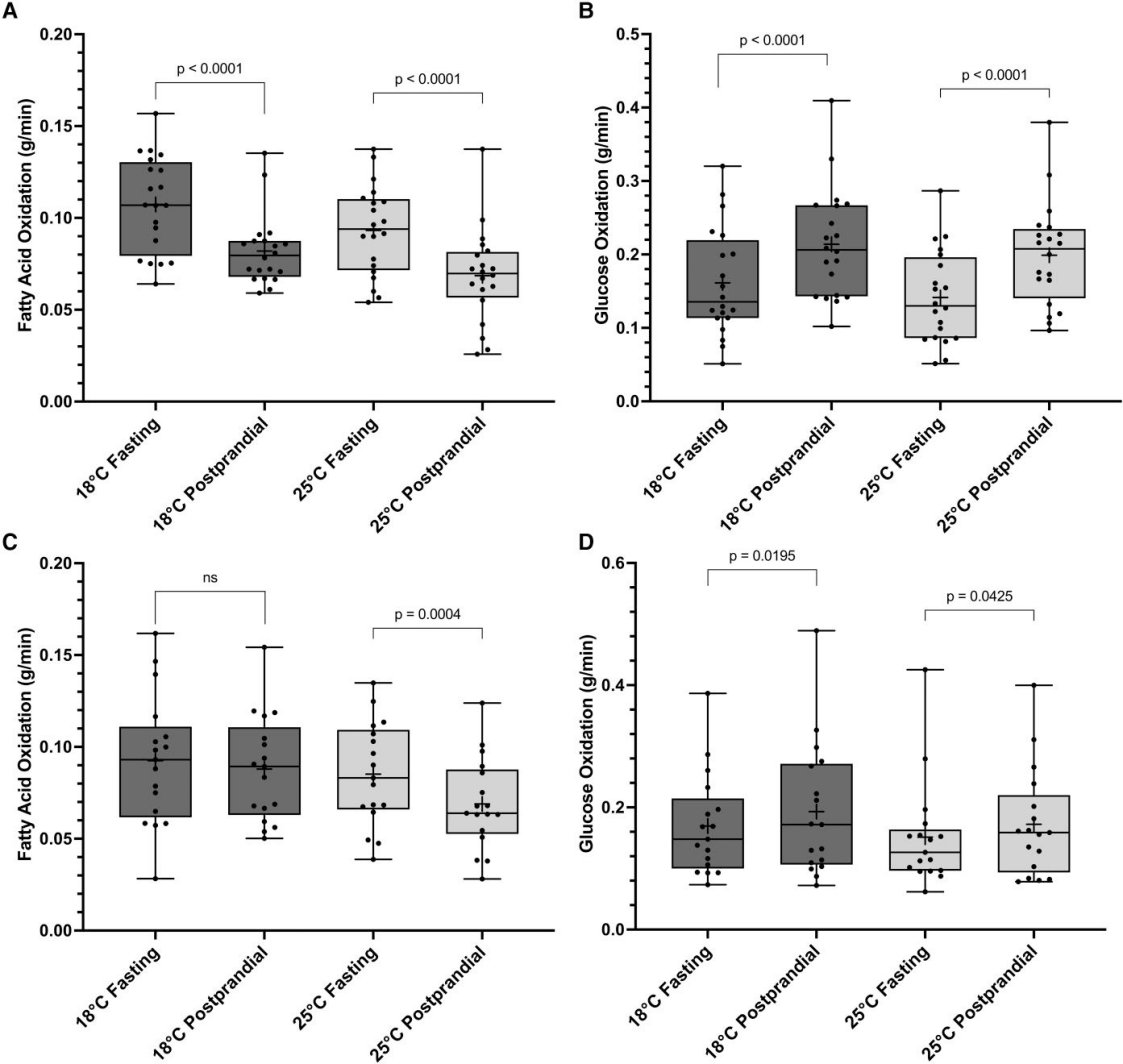
Adult (A) fatty acid and (B) glucose oxidation (g/min) during fasting and postprandial timepoints at room temperature and during cold. Pediatric (C) fatty acid and (D) glucose oxidation (g/min) during comparable fasting and postprandial timepoints. Data shown as box and whisker with min and max values, cross denotes the group mean.

In the fasting state, fatty acid oxidation was similar in both the 25 °C and 18 °C conditions, in both adults and children (Fig. 5). As expected, fatty acid oxidation declined after meal intake (Fig. 5A and 5C). While the magnitude of decline was similar in the 25 °C and cold exposure visits in adults, there was less decline in fatty acid oxidation in the cold condition in children. Glucose oxidation increased after meal intake in adults at both 25 °C (*P* < .0001) and 18 °C (Fig. 5B; *P* < .0001) and in children at the 18 °C (*P* = .02) and 25 °C (Fig. 5D; *P* = .04) visits.

### REE_18_ and BAT activity

**Figure 6:**
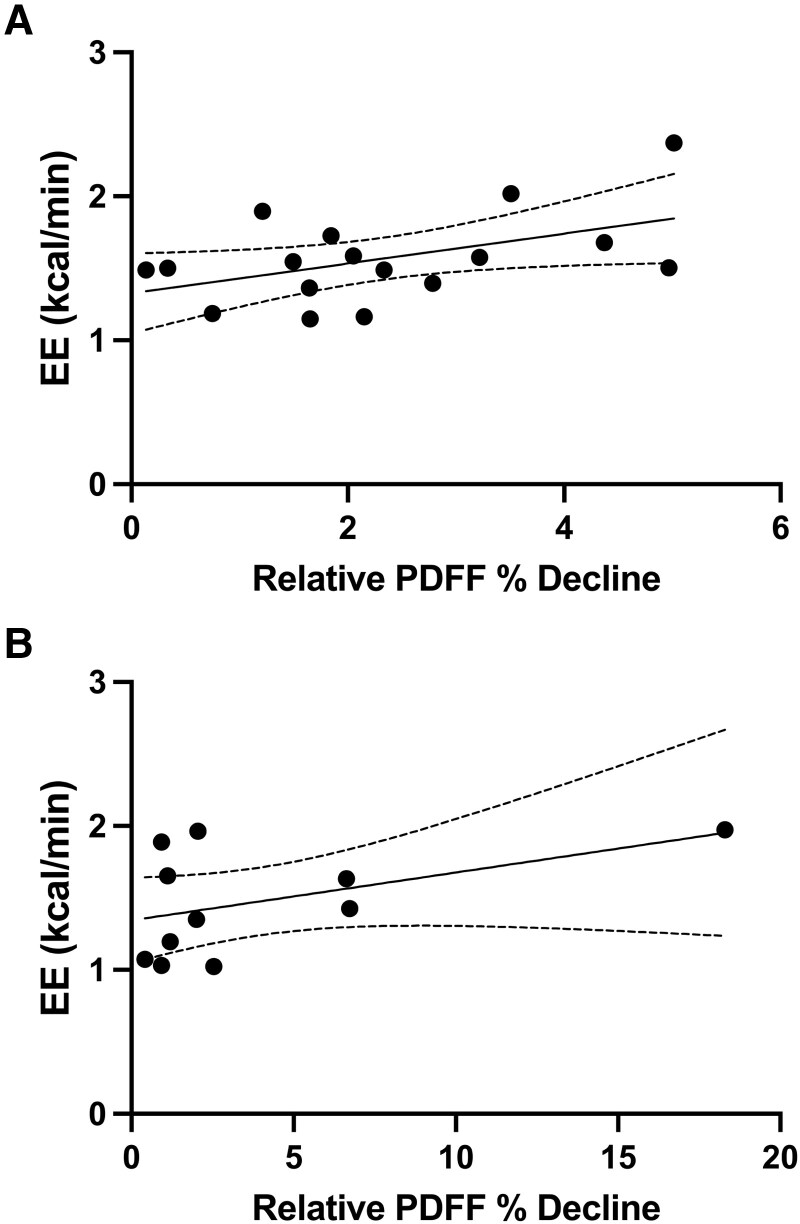
A, Correlation between REE and decline in PDFF among BAT active (n = 17) adult participants (*r* = 0.49, *P* = .046). B, Correlation between REE and decline in PDFF among BAT active (n = 11) pediatric participants (*r* = 0.47, *P* = .14).

The SCV-PDFF declined with cold exposure in 17 of 20 adult participants, suggesting that these participants had active BAT tissue. Among those with evidence of BAT activity, the relative decline in SCV-PDFF with cold was directly related to REE_18_ (*r* = 0.24; *P* = .046) (Fig. 6A), but the relationship was not significant when adult participants with no BAT activity were included (*P* = .58) (Fig. S1) [18].

Of 17 pediatric participants, 14 had MRI scans that could be analyzed for SCV-PDFF (3 had technical issues preventing SCV-PDFF analysis). A relative decline in the PDFF (mean precold PDFF = 65.99 ± 6.9, mean postcold PDFF = 64.24 ± 8.5, *P* = .03) was seen. Among them, 3 of the 14 pediatric participants had no decline in PDFF, suggesting no or low BAT activity. Although the Pearson correlate was similar in children, the relationship between decline in PDFF and REE_18_ (Fig. 6B) in BAT active pediatric participants (*P* = .14) was not significant. In examining the impact of shivering on EE, no significant correlation was found between BAT activity and shivering (adults, *P* = .32; children, *P* = .37). Furthermore, no significant differences were found in EE for participants who reported shivering during cold exposure compared to those that did not.

## Discussion

The aim of the current study was to evaluate the relationship between cold-induced resting EE and BAT activity using a newly established WRIC system, and to explore the feasibility, accuracy and reliability of this system to document changes in EE during cold exposure and after eating a standardized meal in both adults and children. Following the recommendations of the RICORS 1.0 standards for technical and human validation, this study demonstrates that the WRIC system is technically valid and on repeated assessments conducted 1 week apart, it can detect a 10% to 15% increase in EE with cold exposure; also it is feasible to have children as young as 8 years of age in the WRICS for up to 4 hours.

Technical validation of the WRIC was undertaken, adhering to the RICORS 1.0 recommendations. The gas infusion method was utilized during dynamic performance measurement for safety reasons and as it offers greater flexibility in adjusting the infusion rate to alter necessary parameters, an advantage not provided by the gas combustion technique [27]. Previous research studies that have utilized both gas infusion and combustion concluded that gas infusion is a sufficient method [28]. The results of the recovery and RER tests using the gas infusion tests were as expected and consistent with the current literature [28-30].

In evaluating the feasibility of conducting studies in humans as young as 8 years of age, we note the high completion rate in both adults (95%) and children (94%). Although the feasibility of use of a WRICS in adults was similar to previous studies with completion rates of 95% to 98% [29, 31, 32], completion of 94% of children in the present study exceeds many previous studies of children 7 to 18 years of age, where completion varied from 74% to 96% [33-35].

The responsiveness of the system is further demonstrated in its ability to measure changes in EE in response to mild cold exposure. Cold exposure at 18 °C for 2 hours resulted in 0.21 kcal/min (15%) higher EE in adults. In a recent meta-analysis that included 10 studies in healthy adults (total n = 171, aged 20-40 years, BMI 20-34 kg/m^2^), EE increased during cold exposure (varying from 16-19 °C) compared to room temperature. Of these 10 studies, the study by Brychta et al (2019) included the most similar methodology to the present study, including use of whole-room indirect calorimetry to measure EE, and compared room temperatures of 23 to 25 °C as well as 16 °C for the cold exposure, although the cold exposure occurred with changes in ambient temperature and not with a cold-suit as we utilized in the current study [36]. These authors report a 12.5% capacity for cold-induced increases in EE above basal metabolic rate, compared to 15% in the present study. The results of the current study are also in line with estimated predictions from energy cost models based on human heat loss with radiation and convection which predict an increase in REE of 0.1 to 0.4 kcal/min with cold [37]. Increased EE with cold results from increases in both shivering and nonshivering thermogenesis [11]. Shivering thermogenesis, characterized by involuntary muscle contractions, elevates metabolic rate and generates heat [38] whereas nonshivering thermogenesis involves the activation of BAT by the sympathetic nervous system, which plays a crucial role in heat production through the action of uncoupling protein 1 (UCP1) [38, 39]. We identified no difference in the cold-induced increase in EE between those who did and did not report shivering, although shivering was not directly measured with electromyography (EMG). We did identify a direct relationship between BAT activity and REE during cold in adults, with a similar correlation coefficient seen in the smaller number of children that did not reach significance. Our result in adults is consistent with previous work in adults undertaken by Blondin et al (2015) [40] who noted a positive relationship between BAT activity and EE_18_, which increased 1.8-fold following a 180-minute 18 °C cold exposure period using a water perfusion suit. Similarly, Tay et al (2020) [41] reported a positive relationship between BAT activity and EE_18_ as REE increased by 13% following a 45-minute exposure to a room at 18 °C [41].

In contrast to the expected increase in EE in response to cold exposure, no significant increases in EE after eating a standardized meal were demonstrated in either adults or children. The 475-kcal meal was given to all participants, regardless of body size and this may have influenced our findings (ie, insufficient caloric load in larger participants). Other studies have provided a meal with an energy intake based on a standardized percentage of estimated required daily energy intake [42], or as a proportion of measured resting energy expenditure [43, 44]. Given the variability in body size of our participants, the uniform caloric load used in the present study, regardless of individual energy requirements, may have contributed to the blunted thermogenic response observed in both adults and children. A systematic review of studies in adults found that DIT increases by 1.1 kJ/hour (0.004 kcal/min) for every 100 kJ (24 kcal) higher intake, and DIT is also altered by meal composition, especially protein content, which varied in the review [45-47]. Although we did not detect an increase in EE, it is important to note that the WRICS did detect a drop in RER and the resultant increase in glucose oxidation and reduction in fatty acid oxidation consistent with the composition of the meal.

Of note, study participants were not fully reclined or fully resting with eyes closed while REE was being measured. While this has the potential to result in a higher REE, the present study reported mean values of 1.5 to 1.7 kcal/min in adults and children, which is consistent with findings reported in the current literature of 1.2 to 1.7 kcal/min for REE in adults and children [31, 32]. In addition, it is reassuring that measures were reproducible on 2 different occasions, even without this strict limitation on cognitive activity.

In conclusion, in addition to documenting the technical validity of our system, the feasibility of measuring EE in children as young as 8 years of age and the repeatability of REE over a relatively brief period, we have demonstrated increased EE with cold exposure and, in adults the magnitude of this increase is related to BAT activity. These findings provide a strong foundation for future longitudinal, interventional clinical studies.

## Data Availability

Some or all datasets generated during and/or analyzed during the current study are not publicly available but are available from the corresponding author on reasonable request.

## Abbreviations

BAT: brown adipose tissue
BMI: body mass index
DIT: diet-induced thermogenesis
EE: energy expenditure
MASLD: metabolic dysfunction–associated steatotic liver disease
MRI: magnetic resonance imaging
PDFF: proton density fat fraction
REE: resting energy expenditure
RER: respiratory exchange ratio
RICORS: Room Indirect Calorimetry Operating and Reporting Standards
WHO: World Health Organization
WRIC: whole-room indirect calorimetry
WRICS: whole-room indirect calorimetry system
